# Missiles of Mass Disruption: Composition and Glandular Origin of Venom Used as a Projectile Defensive Weapon by the Assassin Bug *Platymeris rhadamanthus*

**DOI:** 10.3390/toxins11110673

**Published:** 2019-11-18

**Authors:** Andrew A. Walker, Samuel D. Robinson, Eivind A. B. Undheim, Jiayi Jin, Xiao Han, Bryan G. Fry, Irina Vetter, Glenn F. King

**Affiliations:** 1 Institute for Molecular Bioscience, The University of Queensland, St. Lucia, QLD 4072, Australia or or e.a.b.undheim@ibv.uio.no (E.A.B.U.); jiayi.jin@uq.net.au (J.J.); x.han2@uq.edu.au (X.H.);; 2 Centre for Biodiversity Dynamics, Department of Biology, Norwegian University of Science and Technology, 7491 Trondheim, Norway; 3 Centre for Ecological and Evolutionary Synthesis, Department of Biosciences, University of Oslo, P.O. Box 1066 Blindern, 0316 Oslo, Norway; 4 Centre for Advanced Imaging, The University of Queensland, St. Lucia, QLD 4072, Australia; 5 Venom Evolution Lab, School of Biological Sciences, The University of Queensland, St. Lucia, QLD 4072, Australia; bgfry@uq.edu.au; 6 School of Pharmacy, The University of Queensland, Woolloongabba, QLD 4102, Australia

**Keywords:** Reduviidae, pacifastin, insecticidal, pain, insect venom, propelled venom

## Abstract

Assassin bugs (Reduviidae) produce venoms that are insecticidal, and which induce pain in predators, but the composition and function of their individual venom components is poorly understood. We report findings on the venom system of the red-spotted assassin bug *Platymeris rhadamanthus*, a large species of African origin that is unique in propelling venom as a projectile weapon when threatened. We performed RNA sequencing experiments on venom glands (separate transcriptomes of the posterior main gland, PMG, and the anterior main gland, AMG), and proteomic experiments on venom that was either defensively propelled or collected from the proboscis in response to electrostimulation. We resolved a venom proteome comprising 166 polypeptides. Both defensively propelled venom and most venom samples collected in response to electrostimulation show a protein profile similar to the predicted secretory products of the PMG, with a smaller contribution from the AMG. Pooled venom samples induce calcium influx via membrane lysis when applied to mammalian neuronal cells, consistent with their ability to cause pain when propelled into the eyes or mucus membranes of potential predators. The same venom induces rapid paralysis and death when injected into fruit flies. These data suggest that the cytolytic, insecticidal venom used by reduviids to capture prey is also a highly effective defensive weapon when propelled at predators.

## 1. Introduction

The practice of spraying or propelling venom against potential predators has evolved in diverse animal taxa including snakes, spiders, ants, wasps, and assassin bugs. Among cobras, projectile use of venom has evolved three times, once in genus *Hemachatus* and twice in genus *Naja* [1]. Spitting cobras propel venom with high accuracy up to 2 m [2], and the venom can cause permanent blindness if it contacts human eyes [3]. Venoms of cobras that are propelled in this way are known to have strong cytotoxic, neurotoxic, and coagulotoxic effects [1,4,5]. Venomous invertebrates that use venom as a projectile weapon are less dangerous to humans but are no less fierce in their microcosm. Workers of the social vespid wasp *Parachartergus colobopterus* [6], formicine ants [7], and the lynx spider *Peucetia viridans* have been reported to propel venom defensively [8]. Spitting spiders (*Scytodes* sp.) uniquely propel a sticky material from their venom glands to immobilise prey mechanically, as well as practising more conventional envenomation [9].

Assassin bugs are predaceous hemipteran insects in the family Reduviidae (excepting the subfamily Triatominae in which a switch to blood-feeding is a derived condition) [10]. The genus *Platymeris* contains species that are particularly large (up to 4 cm), and which have been trialed as biocontrol agents against rhinoceros beetles in coconut palm plantations [11]. More recently, they have become common in the worldwide pet trade. *Platymeris rhadamanthus* and some closely related species are remarkable for being the only reduviids known to propel venom as a defensive weapon when threatened. This is thought to be a defensive adaptation that confers a fitness benefit protecting the slow-moving assassin bugs from insectivorous mammals, birds, and reptiles. Edwards [12] reported the defensive behaviour of *Platymeris* sp., in which bugs respond to physical disturbances and changes in incident light by lifting one side of the body and twisting the unextended proboscis to a point above and behind them; then, a stream of several jets of venom are propelled from the proboscis, up to 30 cm in distance, and containing up to 2 mg dry weight of venom. Sprayed venom was found to have no effect when applied topically to mammals, but produced behaviour consistent with strong pain when applied to the eyes or mucous membranes [12]. Consequently, it was concluded that projectile use of venom represents a defense against vertebrate predators.

Edwards [12] produced a seminal study on the bioactivity of assassin bug venom, also using *Platymeris rhadamanthus* as his study species [13]. During this 1961 study, venom was harvested by inducing animals to propel venom defensively. This venom was found to be potently insecticidal, as injection into cockroaches (*Periplaneta* sp.) caused paralysis within seconds, followed by death. Even a million-fold dilution of venom was sufficient to stop the beating of cockroach hearts, and nerves exposed to diluted venom showed bursts of activity followed by complete loss of function. Histological examination showed that venom rapidly disrupted lipid membranes in the tissue of the injected animal. Thus, Edwards considered the venom of *Platymeris rhadamanthus* to be a single secretion used both for prey capture and predator deterrence [13].

We recently described the venom proteome of the harpactorine assassin bug *Pristhesancus plagipennis* and showed that it comprised numerous enzymes, putative pore-forming toxins and peptides [14]. We also demonstrated that *Pristhesancus plagipennis* produces distinctly different venoms in each of the two compartments of the main venom gland [15]. The posterior main gland (PMG) was found to produce venom that quickly paralyses and kills prey insects. The smaller anterior main gland (AMG) was found to produce a complex venom with very different composition that showed less pronounced effects when injected into insects. Since venom made in the AMG could be elicited by harassment, we proposed that AMG venom may have a role in predator deterrence, or alternately have antimicrobial or anticlotting activities. These results differ from a report that the AMG produces insecticidal neurotoxins whereas the PMG produces digestive enzymes [16], as well as Edwards’ report that *Platymeris rhadamanthus* AMG and PMG gland extracts show similar paralytic activity [13]. Moreover, the glandular origin of venom propelled defensively by *Platymeris rhadamanthus* in response to harassment has remained unknown.

Here, we address these questions by using transcriptomics, proteomics, and functional assays to investigate the venom system of *Platymeris rhadamanthus*. We determine that numerous patterns of toxin gene expression observed in *Pristhesancus plagipennis* also are observed in *Platymeris rhadamanthus*. However, the two species show key differences in how the products of each gland could be collected in a laboratory setting. These data provide insights into how reduviid venom, which is usually injected either offensively during hunting, or defensively against potential predators, has been adapted as a projectile weapon.

## 2. Results

### 2.1. Venom Proteome

*Platymeris rhadamanthus* adults yielded large volumes of venom (often >10 µL per individual) in response to electrostimulation (20 V constant DC) applied across the thorax. Some individuals also displayed defensive behaviour similar to that previously reported [12] wherein a short series of venom drops is expelled from the curved, unextended proboscis and is sprayed ~30 cm beyond the rear of the insect.

To determine the composition of *Platymeris rhadamanthus* venom, we combined venom gland transcriptomics with venom proteomics. To generate venom gland transcriptomes, the PMG and AMG were dissected out separately and Poly(A)+ RNA from each tissue sequenced, resulting in 42,324,442 and 67,317,010 reads, respectively. Reads from both the PMG and AMG were assembled together to produce one Trinity assembly and six CLC Genomics Workbench assemblies (see Materials and Methods). Translated open reading frames >30 amino acids were extracted using TransDecoder and used (together with a decoy database of common protein contaminants) as a search database against which liquid chromatography-tandem mass spectrometry (LC-MS/MS) data obtained from venom samples could be used to identify venom proteins. The venom proteome we report (Supplementary Data S1, GenBank accessions MN208278–MN208442) is the result of searching all venom samples analysed in this study against this sequence database. These samples comprised reduced, alkylated and trypsinised venom obtained by electrostimulation (17 samples), or venom that was defensively propelled from the proboscis (3 samples), plus 128 gel spots that are described in more detail in Section 2.3.

Despite representing a different subfamily of assassin bugs, *Platymeris rhadamanthus* showed a similar venom profile to that we reported for *Pristhesancus plagipennis*. The major classes of polypeptides detected in the venom were S1 proteases (66), peptides (18), members of heteropteran venom protein family 2 (15), redulysins (14), hemolysin-like proteins (7), and proteins consisting of a single CUB (complement component 1r:1s/urinary epithelial growth factor/bone morphogenic protein 1) domain (6). Only two proteins were detected that displayed no homology to known sequences.

Two identified venom components showed very high similarity to polypeptides reported from the venom of a congeneric species, *Platymeris biguttatus*, that were sequenced by Edman degradation and PCR-based methods (Supplementary Figure S1A,B) [17]. *Platymeris rhadamanthus* Redulysin 2 is 92% identical to Platylysin, a protein detected in a semi-pure insecticidal fraction of *Platymeris biguttatus* venom [17]. *Platymeris rhadamanthus* peptide Pr5a is a member of the Ptu1 family of disulfide-rich knottins and it differs from the *Platymeris biguttatus* peptide Pb1a by only a single conservative change (Lys rather than Arg at position 13 of the mature peptide) [17]. Another *Platymeris rhadamanthus* venom component, CUB domain protein 1, displays Basic Local Alignment Search Tool (BLAST) homology (*E* = 2 × 10^–10^) to triatox (Supplementary Figure S1C), a venom component of unknown function from venom of the blood-feeding reduviid *Triatoma infestans* [18] that we have previously suggested is a member of the CUB domain family [19].

Other components of the *Platymeris rhadamanthus* venom proteome previously have not been reported from assassin bugs and are likely unique to this species and some subset of related reduviids. Three proteins we named Redulysin-like proteins 1–3 show BLAST homology to the *N*-terminal prodomains of redulysins, for example, but lack the *C*-terminal domain. Additionally, a high number of amino acid sequences (eight protein precursors of 2–5 domains each) were detected that show homology to the disulfide-rich repeats of the light chain of pacifastin, a 155 kDa serine protease inhibitor from the crustacean *Pacifasticus leniusculus* [20].

### 2.2. Conserved Patterns of Venom Gland Specialisation in Assassin Bugs

**Figure 1:**
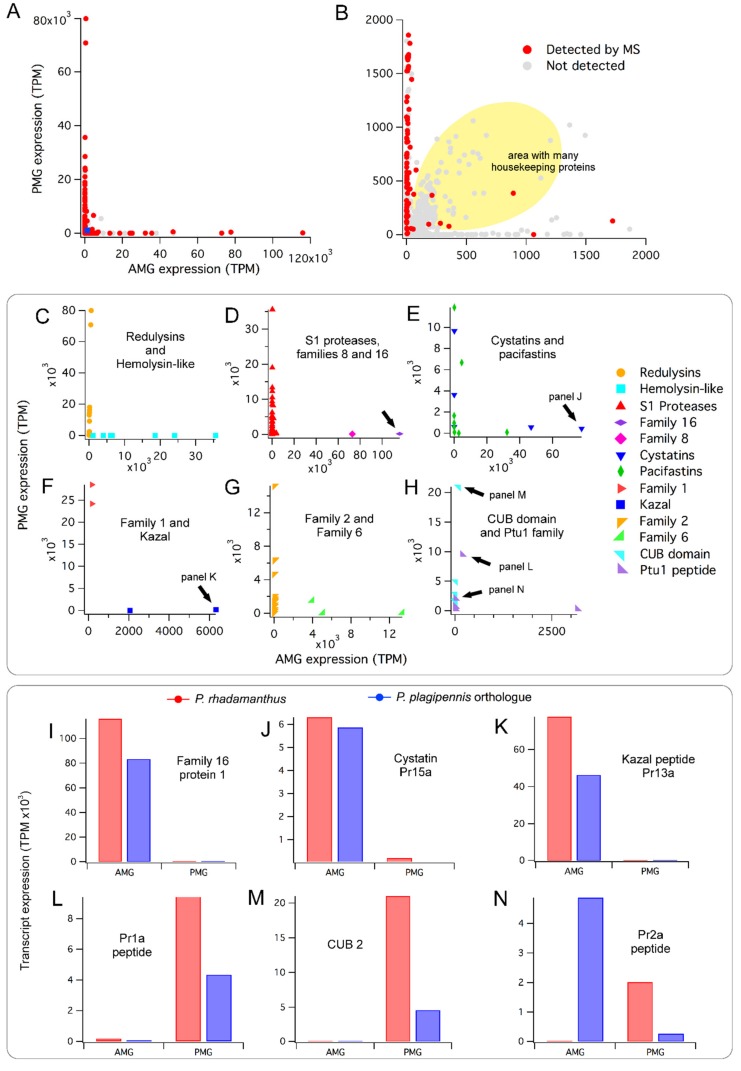
Gland-specific toxin expression (previous page). (**A**) Venom proteins detected by mass spectrometry (MS) are highly expressed in the posterior main gland (PMG) or anterior main gland (AMG) but not both. TPM, transcripts per million. (**B**) Enlargement of area represented by the small blue square in bottom left of panel A. The yellow area contains many putative housekeeping proteins expressed in both the PMG and AMG. (**C**–**H**) Comparison of expression values for individual venom polypeptide families. CUB domain, complement 1r:1s/urinary epithelial growth factor/bone morphogenic protein 1 domain; Ptu1 peptide, member of the family of knottins discovered in venom of the assassin bug *Peirates turpis* [21] (**I**–**N**) Comparison of gland-specific expression for putative orthologue pairs between reduviine *Platymeris rhadamanthus* and harpactorine *Pristhesancus plagipennis*. Panel I corresponds to the protein indicated by an arrow in panel D; panel J to that in panel E; K to that in panel F; and L–N to those indicated in panel H. Expression values for *Pristhesancus plagipennis* are from Walker et al. [15].

To obtain estimates of transcript abundance in each of these venom glands, we mapped reads obtained from RNA purified from each of the PMG and AMG against assembled contigs to obtain transcripts per million (TPM) values (see Materials and Methods). We found that most of the polypeptides detected in venom using mass spectrometry (MS) are encoded by transcripts which are highly abundant in at least one of the two gland regions (Figure 1, Supplementary Data S1). However, the pattern of expression values obtained showed a highly non-random distribution, with individual transcripts being highly expressed (>10000 TPM) in one, but not both of the PMG and AMG (Figure 1A). This result is perhaps surprising, since house-keeping proteins would presumably be required by both tissues regardless of what proteins they secrete. Further examination revealed the expected pattern of house-keeping proteins expressed at similar values in both glands, but at much lower transcript abundances of 10–2000 TPM (Figure 1B; Supplementary Data S2). BLAST searches of a randomised subset of the shaded region in Figure 1B infer that >50% of these encoded non-venom housekeeping proteins such as ribosomal proteins and histones. To summarise, we see that venom transcripts are highly expressed and almost always seen in one, but not both, of the two glands, while transcripts encoding house-keeping proteins show lower expression and typically are expressed close to evenly across the two glands.

Since only one biological RNA-Seq replicate was performed, we did not analyse preferential expression of single transcripts, but instead used the Kruskal–Wallis statistic to test if preferential expression occurred at the level of families of multiple homologous proteins detected in venom by MS. Protein families preferentially expressed in the PMG included redulysins (Figure 1C; Kruskal–Wallis test *p* < 10^−3^), S1 proteases (Figure 1D; *p* < 10^−18^), heteropteran venom family 2 (Figure 1G; *p* < 10^−5^), CUB domain proteins (Figure 1H; *p* < 0.005), and Ptu1 family peptides (Figure 1H; *p* < 0.05). The AMG preferentially expresses hemolysin-like proteins (Figure 1C; *p* < 0.005), and heteropteran venom family 6 proteins (Figure 1G; *p* < 0.05). Families 16 and 8, as well as Kazal domain peptides in the AMG, and family 1 in the PMG, showed very different transcript abundance estimates for the two glands (Figure 1D,F), but did not contain a sufficient number of members to analyse statistically. Contigs encoding cystatins and pacifastins were strongly expressed in both venom glands, but individual proteins showed high and gland-specific expression in only one of the two glands (Figure 1E).

To further compare transcript abundance values between *Platymeris rhadamanthus* and *Pristhesancus plagipennis*, we compared their venom gland transcriptomes using reciprocal BLAST searches, revealing 57 pairs of putative orthologues, each member of which is confidently identified in venom by MS (Supplementary Data S3). 51 of these orthologue pairs (89%) showed a similar pattern of transcript abundance values between the AMG and PMG in both species (e.g., Figure 1I–M), whereas only six have contrasting patterns (e.g., Figure 1N). Although additional biological replicates would be required to confirm these data, they suggest conservation of numerous venom system traits between these two assassin bug species, and support our previous reports of venom-gland specialisation in other heteropteran species [15,19].

Comparison of transcriptomic data with proteomic data suggests that MS was able to detect most of the secretion products of the PMG, and some of the products of the AMG. All 37 transcripts with high transcript abundance values (>5000 TPM) in the PMG that begin with a predicted secretion signal peptide and end with a stop codon encode a venom protein that was detected by MS. A large number of proteins preferentially expressed in the PMG with much lower transcript abundance (10–2000 TPM) were also detected (Figure 1B). Regarding proteins highly expressed in the AMG (TPM >5000), 17 of 24 (71%) were detected by MS, whereas only a smaller proportion of transcripts with lower AMG transcript abundances (10–2000 TPM) were detected (Figure 1B).

### 2.3. Major Proteins and Peptides in Venom Obtained by Electrostimulation

To investigate if venom samples expelled in response to electrostimulation differed in their composition or likely glandular origin to venom samples propelled defensively, we compared venom collected by each method using proteomics. Venom obtained by electrostimulation, which was available in large quantities, was used to perform both LC-MS/MS experiments aiming to resolve the mature form of peptides, and two-dimensional polyacrylamide gel electrophoresis (2D-SDS-PAGE) aiming to better resolve the abundance of larger proteins. Pooled venom from multiple samples obtained by electrostimulation were used for these experiments.

Comparison of LC-MS/MS data from venom obtained by electrostimulation before and after reduction/alkylation (but without trypsin digestion) yielded the mature forms of 12 peptides present in venom (Table 1). Most of these (11 of 12) contained six cysteine residues and, of these, six peptides possessed a cysteine pattern suggesting they are members of the Ptu1-like knottin family [14,21,22].

**Figure 2:**
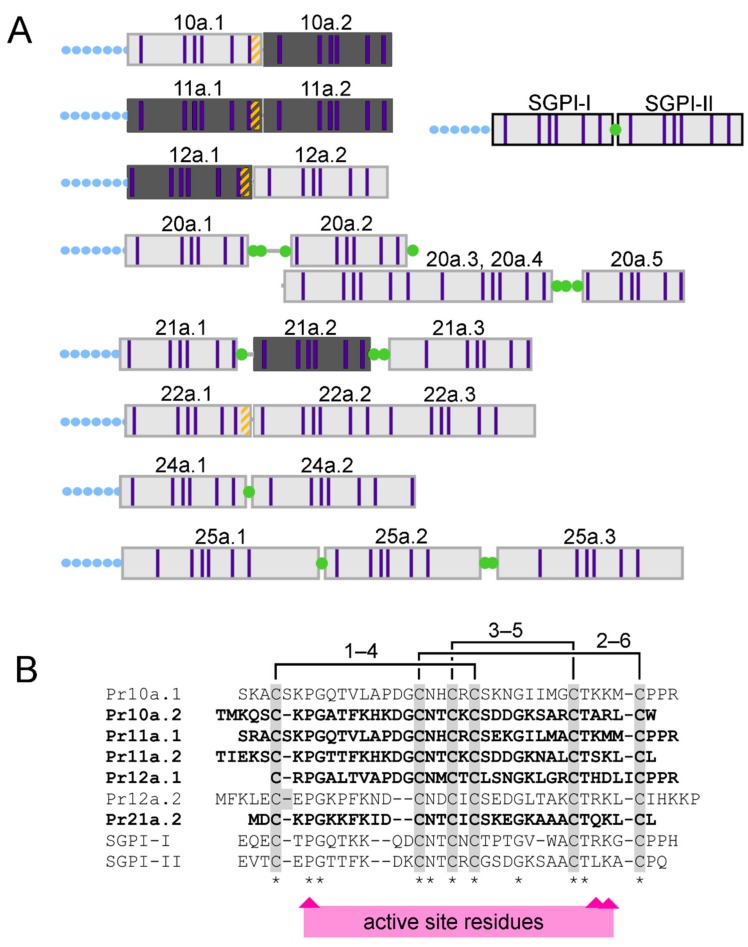
Processing of pacifastin venom peptides. (**A**) Domain architecture of pacifastin precursors with *Schistocerca gregaria* protease inhibitors I and II (SGPI-I and SGPI-II) for comparison. Green dots indicate dibasic sites similar to those cleaved during SGPI processing; orange stripes denote Pro-Pro-Arg sites that we found are cleavage sites for proteolytic processing of *Platymeris rhadamanthus* pacifastin; blue-dotted lines indicate secretion signal sequences; and purple bars denote cysteine residues. (**B**) Alignment of *Platymeris rhadamanthus* venom pacifastins with SGPI peptides. The disulfide connectivity of SGPI peptides is shown above the sequences. Residues critical for inhibition of serine proteases by SGPI peptides are indicated below the sequence alignment. Darker-shaded peptides in panel A, and sequences in bold in panel B, were detected in their mature form in MS analysis of untrypsinised venom samples.

The remaining five disulfide-rich peptides, each with molecular mass 3.7–4.2 kDa, corresponded to a single domain of a multidomain pacifastin precursor (Figure 2; see Section 2.1). Comparison of the mature primary structures with the venom-gland transcriptomic data revealed that the single-domain pacifastin venom peptides were derived by proteolytic processing of a larger precursor, similar to the ‘multifunctional warhead’ precursors we described in centipede venom systems [23]. Although we previously detected polypeptides with pacifastin-like disulfide-rich repeats in the venoms of harpactorine assassin bugs [14,15], these consisted of much longer multidomain repeat precursors, and the mature form of the polypeptides are unknown. Indeed, the *Platymeris rhadamanthus* pacifastin peptides Pr10a–Pr12a and Pr20a–Pr24a were not recovered as orthologous to pacifastin domains previously detected in assassin bug venom (Supplementary Data S3) and, instead, show greater BLAST homology and more similar precursor architecture to peptides of ~4 kDa that have been isolated from orthopteran insects such as *Locusta migratoria* and *Schistocerca gregaria* [24,25,26]. Whereas almost all known insect single-domain pacifastin peptides are processed by cleavage at dibasic sites [27], our transcriptomic and MS data indicated that many *Platymeris rhadamanthus* venom pacifastins are cleaved at monobasic sites, especially following Pro-Pro-Arg sites such as those that occur in Pr10a, Pr11a, and Pr12. In contrast to dibasic pacifastin cleavage sites, these PPR sites were observed to be retained at the *C*-terminus of the mature peptides U-RDTX-Pr11a.1 and U-RDTX-Pr12a.1 (Figure 2). Residues reported to be important for serine protease inhibition activity [27] showed variable conservation, making it unclear if these peptides are functional serine protease inhibitors or performed other roles.

**Figure 3:**
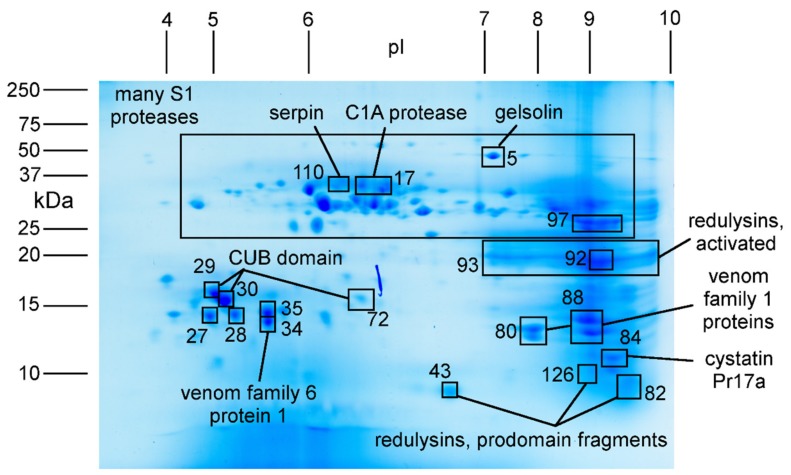
2D-SDS-PAGE gel of *Platymeris rhadamanthus* venom. Boxed squares show gel spots from which clear protein identifications were obtained by LC-MS/MS. Isolectric point (pI) is shown at the top and mass (in kDa) is shown on left. Numbers show spots described in Table 2.

To examine the abundance and mature form of larger proteins, we separated venom components according to their isoelectric point (pI) and mass using 2D-SDS-PAGE. Following staining, 128 gel spots were excised and subjected to protein identification experiments using LC-MS/MS (Figure 3). This process revealed the most abundant components of venom obtained by electrostimulation to be S1 proteases, redulysins, CUB domain proteins, cystatin Pr17a, the actin depolymerising protein gelsolin, and members of heteropteran venom protein families 1 and 6 (Figure 3). These proteins were all strongly expressed in the PMG (Figure 1).

More detailed analysis revealed additional information about the mature forms of the redulysin family proteins. Although the redulysins (after removal of their secretion signal sequences) were predicted to have masses of 23–70 kDa and pI values from 5–9.5, we obtained the strongest signal for redulysin peptide fragments from an intensely stained gel region corresponding to pI 7–10 and mass 18–22 kDa (Figure 3, Table 2). Moreover, tryptic fragments obtained from this gel region were derived only from the region of each protein that was *C*-terminal to the Asp-Glu-Glu-Arg (single letter code, DEER) site that was reported to be a prodomain cleavage site in the homologous trialysin protein from blood-feeding reduviids [28]. Cleavage at this site of trialysin was proposed to remove the anionic prodomain and expose the lysine-rich lytic domain, thereby activating the protein [29]. This site is strongly conserved in *Platymeris rhadamanthus* redulysins (primary structure DEER in redulysins 1–8 and 11, and NEER and NEELGR in redulysins 10 and 9, respectively). Subsequent to removal of the prodomain, these putative active forms of the redulysins were predicted to have a molecular mass of 21–23 kDa (or 29 kDa in the case of redulysin 1) and a pI of 9.1–9.6, closely matching the intensely stained region of the gel from which the protein identifications were obtained. Although tryptic fragments derived from redulysin prodomains also were detected, these were from separate, lower molecular mass spots (Figure 3). These data suggested that in the venom sample analysed, redulysins exist principally in their ‘activated’ forms with the prodomain removed. Possibly, the DEER site was cleaved by venom proteases upon venom extrusion or in the lumen of the venom gland, or within the secretory pathway.

### 2.4. Composition and Glandular Origin of Defensively Propelled Venom

**Figure 4:**
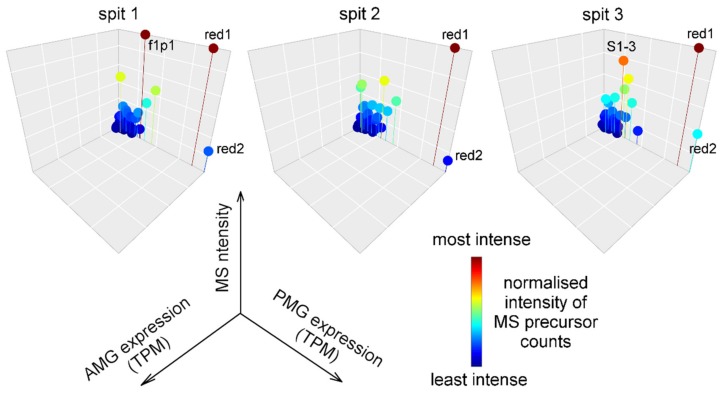
Venom propelled defensively originates from the posterior main gland. The three plots represent tandem mass spectrometry (MS) analysis of three propelled venom samples from three different individuals. Each marker represents a particular venom protein or peptide. Gridlines on the *x* and *y* axes represent 20000 transcripts per million (TPM), and on the *z* axis 20% of maximum. Labels red1, Redulysin 1; red2, Redulysin 2; f1p1, Venom protein family 1 protein 1; S1-3, S1 protease 3.

Since we previously demonstrated that harassment of *Pristhesancus plagipennis* elicits the secretory products of the AMG (but not the PMG), we hypothesised that venom propelled defensively by *Platymeris rhadamanthus* might be partly or wholly produced by the AMG. To test this, we used LC-MS/MS to analyse three samples of venom defensively propelled by three individual bugs. However, we did not find any support for this hypothesis. All three samples of propelled venom primarily contained proteins that had higher estimated transcript abundances in the PMG compared to the AMG (Figure 4). This trend was highly statistically significant (Kruskal–Wallis test on TPM values of detected proteins in PMG and AMG gives *p* < 10^−19^ in each case). All three samples showed maximum protein signal originating from redulysin 1. These results suggest that venom propelled defensively was produced in the PMG.

### 2.5. Likely Glandular Origin of Venom Obtained by Electrostimulation.

**Figure 5:**
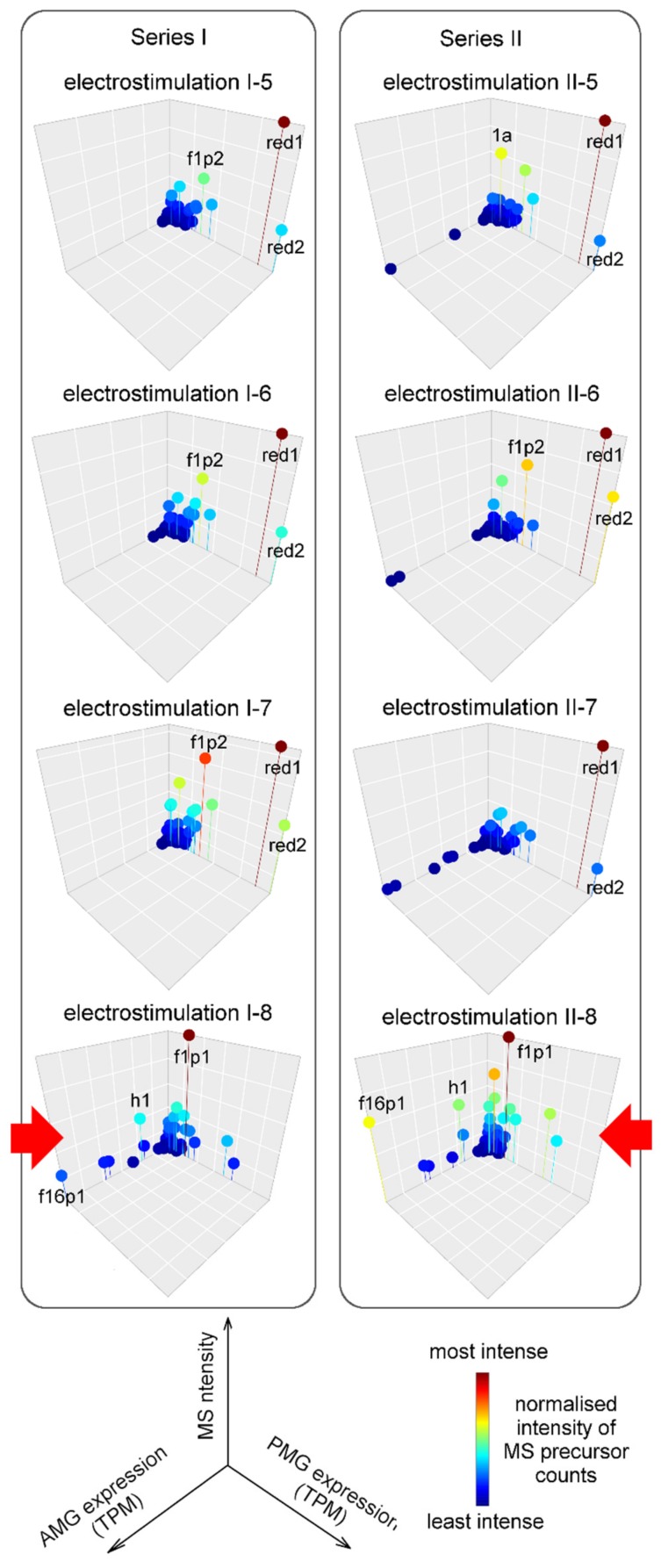
Glandular origin of venom harvested by electrostimulation. Two series of venom samples collected from single individuals consecutively over the course of a single venom harvest. Tandem mass spectrometry (MS) analysis of the final four samples of each series are shown. Gridlines on the *x* and *y* axes represent 20000 transcripts per million (TPM), and on the *z* axis 20% of maximum. Labels red1 and red 2 indicate Redulysins 1 and 2; f1p1 and f1p2, Heteropteran venom protein family 1 proteins 1 and 2; 1a, peptide Pr1a; h1, Haemolysin-like protein 1; f16p1, Heteropteran venom protein family 16 protein 1. Red arrows show final sample with composition differing from those previous.

Since proteins that our transcriptomic data suggested were highly expressed in the AMG were not detected in propelled venom samples, we analysed additional venom samples individually to determine from which venom samples these protein identifications were obtained. The samples we analysed included two series of eight venom samples collected consecutively from single animals (Figure 5). Most samples obtained by electrostimulation either lack (Figure 5, Series I) or contain only small amounts (Figure 5, Series II) of proteins that are highly expressed in the AMG. Surprisingly, the final sample obtained in each series was enriched in such proteins (Figure 5, bottom panels) compared to previous samples in the series, and showed comparable signals for proteins preferentially expressed in each of the two venom glands. These results contrast with our previous findings on another reduviid, *Pristhesancus plagipennis* [15], and suggest that, although the secretory products of each of the PMG and AMG are highly conserved among reduviids (Section 2.2), the conditions under which the contents of each gland can be obtained in the laboratory are variable between species.

### 2.6. Venom Activates Mammalian Nociceptors and Kills Insects

Since propelled venom was found to be similar in composition to most venom samples obtained by electrostimulation, we hypothesised that this venom may show bioactivity consistent with the ability to induce nociception when propelled defensively. To test this hypothesis, we performed imaging experiments that detected changes in intracellular calcium concentration ([Ca^2+^]_i_) in mammalian sensory neurons. An increase in [Ca^2+^]_i_ serves as a proxy for pain induction as the final event in activation of nociceptive neurons. This assay was used previously to characterise the nociceptive activity of venoms [30,31,32]. To conduct these experiments, we used pooled venom from multiple samples obtained by electrostimulation (the same pool used in Section 2.3).

**Figure 6:**
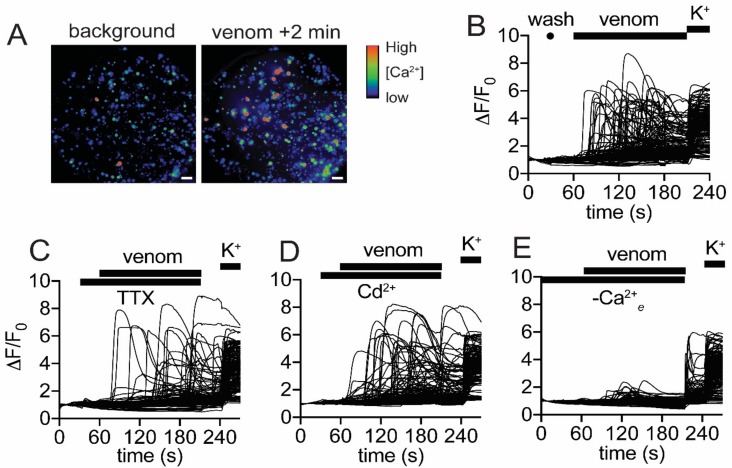
Venom causes rapid calcium influx in mammalian sensory neurons. (**A**) Mouse dorsal root ganglion (DRG) cells loaded with calcium-sensitive dye before (background) and 2 min after addition of venom (100 ng/µL). Scale bars are 200 µm. (**B**–**E**) Effect of venom on DRG cells in the presence of (**B**) Regular assay buffer; (**C**) 1 µM tetrodotoxin (TTX); (**D**) 100 µM Cd^2+^; and (**E**) in the absence of extracellular Ca^2+^. Replacement of the buffer without venom at 30 s (marked “wash” in panel B) served as a negative control. Addition of KCl at the end of each experiment served as a positive control to identify live neurons. Traces represent individual cells, and each panel corresponds to one representative experiment.

Figure 6A–E illustrate the effect of propelled venom on mouse dorsal root ganglion (DRG) cultures. Increases in [Ca^2+^]*_i_* occurred in all cells present in the culture, with a rapid rise after a variable delay (Figure 6B). The increases in [Ca^2+^]*_i_* invariably were followed by a gradual decrease back to baseline, and this was due to visible leakage of the Ca^2+^-sensitive dye from the cells into the extracellular medium. This response was maintained in the presence of either 1 µM tetrodotoxin (TTX) or 100 µM Cd^2+^ (Figure 6C,D), indicating that voltage-gated sodium and calcium channels, respectively, were not necessary for the observed increases in [Ca^2+^]*_i_*. However, cellular responses largely were attenuated in the absence of extracellular Ca^2+^, indicating that the increase in [Ca^2+^]*_i_* is due to flux of Ca^2+^ across the plasma membrane, rather than release from intracellular stores. Taken together, these data suggest that the observed effect of venom was due to pore formation and/or disruption of cell membranes that allows both influx of extracellular Ca^2+^ and efflux of the indicator dye (Fluo-4-AM, 737 Da) and probably other cellular contents. Our results were consistent with venom causing pain when defensively propelled into the eyes or mucous membranes of potential predators [12], since membrane disruption was likely to cause depolarisation of nerve endings as well as localised tissue damage to generate nociceptive signalling.

**Figure 7:**
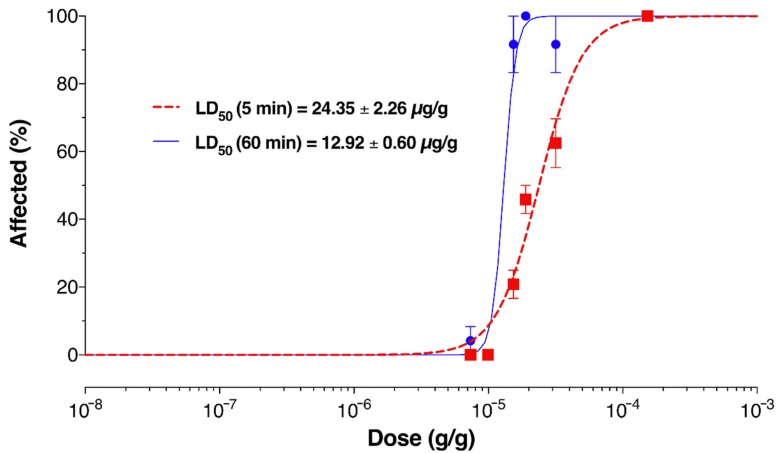
Insecticidal activity of venom. Dose dependence of lethal effects of venom injection measured 5 min (red dashed trace) or 60 min (blue solid trace) after injection. The effect of each dose of venom was tested by injecting three groups of eight flies. Data points are mean values, and error bars indicate the standard deviations between groups.

To test if the same venom had effects on invertebrates that could facilitate prey capture during hunting, we injected venom into fruit flies (*Drosophila melanogaster*). Injection of 0.4 µg venom into the haemolymph of each of 24 adult female flies induced, in every case, rapid flaccid paralysis followed by death, with an LD_50_ (dose required to kill 50% of flies) of 12.92 ± 0.60 µg/g at 1 h after injection (Figure 7, blue trace). The LD_50_ measured at 5 min after injection was only slightly higher, 24.35 ± 0.60 µg/g, indicating the rapid action of the venom. Thus, our data indicated that venom collected by electrostimulation, which contains secretory products mainly from the PMG and to a smaller extent from the AMG, was likely to be effective both when propelled in defense and when injected during hunting.

## 3. Discussion

We used proteotranscriptomics and functional assays in this study to investigate the composition, function, and glandular origin of venom of the reduviine assassin bug *Platymeris rhadamanthus*. Most polypeptides detected in venom belong to families that have been identified previously in assassin bug venom [14,15,17,21]. These data support our previous observations that the composition of heteropteran venom is highly conserved through evolutionary time in the absence of trophic transitions [19], a feature that has been noted for some other groups of venomous animal [33]. Functional assays suggest that *Platymeris rhadamanthus* venom has cytolytic activity which underlies its use as a defensive projectile weapon against predators, and paralytic/insecticidal activity that underlies its use as an offensive weapon while hunting.

One novel observation of this study is the detection of single-domain pacifastin peptides in *Platymeris rhadamanthus* venom with molecular masses close to 4 kDa. Pacifastin domain peptides have been reported in venom of the parasitoid wasp *Nasonia vitripennis* [34], and they have been suggested to be important components of coleoid (squid, cuttlefish, and octopus) venoms [35]. The *N. vitripennis* venom pacifastin has been shown to inhibit prophenoloxidase [34], an enzyme essential for both clotting and insect innate immune responses [36]. *Platymeris rhadamanthus* pacifastin peptides might have similar activity and facilitate assassin bug feeding by preventing the clotting of prey haemolymph. Alternatively, they may have activity similar to disulfide-rich peptides from other animal venoms that modulate neuronal ion channels to induce paralysis or pain, thereby facilitating prey capture or defense [37,38].

We analysed our proteotranscriptomic data to test if venom obtained by inducing defensive venom propulsion or by electrostimulation differed in composition, and to investigate the glandular origin of venom. We found evidence that *Platymeris rhadamanthus* shows several differences compared to previously reported results with regard to how it reacts to stimuli intended to elicit venom in the laboratory. However, questions remain surrounding the biological role of the two venom glands, the AMG and the PMG.

Sequencing of RNA purified from the PMG and AMG revealed patterns of estimated transcript abundance that closely mirror those we reported for other heteropterans [15,19]. Although these data are based on a single sequencing replicate and therefore probably contain artefacts due to uncontrolled individual and temporal variations, we consider that some of the trends we observed are likely to reflect biologically relevant patterns in gene expression, for the following reasons:

(1) The differences are very large. Most (113 out of 166, 68%) of the polypeptides detected in this study showed estimated transcript abundances that were >100-fold higher in one gland or the other.

(2) The distribution of transcript abundances (Figure 1, panels A and B) shows a highly non-random distribution in which many venom proteins are highly expressed in one, but not both, glands.

(3) The patterns of estimated transcript abundance are highly similar to the patterns reported for other venomous heteropterans. Two of these studies used venom gland proteomics as well as transcriptomics to examine protein abundance in each of the PMG and AMG [15,19]. The data obtained in this study suggests that in *Platymeris rhadamanthus*, redulysins, S1 proteases, members of heteropteran venom families 1 and 2, CUB domain proteins, and Ptu1 family peptides are most highly expressed in the PMG, whereas hemolysin-like proteins, members of heteropteran venom families 6, 8, and 16, and Kazal domain peptides, are most highly expressed in the AMG. These patterns are highly similar to those observed in studies of other heteropteran species that also have incorporated proteomic data [15,19]. The simplest explanation for these trends is that they reflect conservation of many features of the venom system over the ~65 million years of evolution since the lineages leading to *Platymeris rhadamanthus* and *Pristhesancus plagipennis* diverged [39].

We did not find any functional evidence supporting the specialisation of the two glands. Although we hypothesised that venom propelled during defensive behaviour would have a different composition to venom obtained by electrostimulation and might be derived from the AMG, we found little or no evidence to support these hypotheses. Instead, propelled venom samples were similar in composition to venom obtained by electrostimulation, and both more closely resembled the products of transcripts expressed more strongly in the PMG. Although we could detect some venom proteins that are highly expressed in the AMG, especially among the final samples in consecutive series obtained by electrostimulation, it is hard to draw a conclusion relevant to reduviid biology from this finding. However, these results are consistent with previous observations that different assassin bug species respond in highly variable ways to stimuli designed to elicit venom in the laboratory [40].

The pooled venom obtained by electrostimulation that we tested functionally showed strong cytolytic effects when applied to mammalian sensory neurons, and strong insecticidal effects when injected into insects. These strong cytolytic effects of venom make it suited for defense as a projectile weapon when launched at the eyes and mucous membranes of vertebrates. Direct injection of this venom into vertebrates is likely to be painful, and this cytotoxic effect may also underlie the painful effect of venom used defensively but directly injected by other reduviid species. A limitation of this study is that, although it provides information about the bioactivity and composition of venom, it does not identify which venom toxins are responsible for the observed effects. Highly abundant venom proteins, such as redulysins 1 and 2, might be the components responsible for the observed disruption of sensory neuron membranes, since they are homologous to proteins such as trialysin, which forms pores in lipid bilayers [28], but this requires further investigation.

The same venom was potently insecticidal when injected into *Drosophila*, indicating it is highly suited both for offensive use during hunting as well as use as a defensive weapon against vertebrate predators. The LD_50_ obtained, 12.92 µg/g 1 h after injection, is very close to the value previously reported for injection of *Platymeris rhadamanthus* venom into the haemolymph of cockroaches (*Periplaneta* sp.), 10.25 µg/g [12] and is similar to values for scorpion and spider venoms injected into insects [41]. It will be interesting to determine what toxins are responsible for the rapid insecticidal activity of venom, and if these are related to the observed cytolytic bioactivity. Although rapid paralysis is usually associated with peptide or small molecule toxins, paralytic pore-forming toxins also have been reported. The 27 kDa hydralysin produced by the cnidarian *Chlorohydra viridissima,* for example, induces rapid paralysis in prey through a cell-type specific, receptor-mediated pore-forming process [42], suggesting that pore-forming toxins may underlie prey paralysis induced by some types of venom. Aculeatoxins such as the peptide Mg1a from the bull ant *Myrmecia gulosa,* also are examples of peptides that have paralytic effects but act directly on plasma membranes [30].

Future studies employing methods such as assay-guided fractionation of crude venom combined with recombinant expression or synthetic production of individual toxins will be required to identify the insecticidal and algogenic components of *Platymeris rhadamanthus* venom, and to clarify what role the AMG plays, if any, in reduviid envenomation. This study reports, for the first time, a detailed account of the venom polypeptides produced in the venom glands of *Platymeris rhadamanthus*. The information we obtained on venom polypeptide primary structures via integration of proteomic and transcriptomic studies should provide a valuable platform for future identification and characterisation of bioactive peptides and proteins from reduviid venoms.

## 4. Materials and Methods

### 4.1. Insects and Venom Collection

Adult *P. rhadamanthus* were purchased from invertebrate supplier Virginia Cheeseman (www.virginiacheeseman.co.uk). Venom was collected by electrostimulation (constant voltage 20 V) or by inducing venom spitting with bugs semi-contained, as we described elsewhere [40]. Several microlitres of protease inhibitor cocktail (cOmplete protease inhibitor, EDTA free, #4693132001, Roche, Basel, Switzerland) was added to venom samples, which were then lyophilised. Following resuspension, venom samples were stored at −20 °C.

### 4.2. Transcriptomics

Two individuals were anaesthetised with CO_2_ for 10 min, and their main venom gland complexes dissected out and stored in >10-fold of the tissue volume of RNAlater. The PMG and AMG were readily distinguishable by their size and appearance. Each gland type, from each side of the body and from both individuals, were pooled into different tubes. Total RNA was extracted using an RNeasy kit (#74104, Qiagen, Hilden, Germany) from the PMG (10 mg wet weight) and AMG (2 mg wet weight), according to the manufacturer’s instructions. This resulted in 110 µg (PMG) and 13 µg (AMG) of total RNA. Poly(A)+ mRNA was purified using a Dynabeads mRNA Direct kit (#64011, Thermo Fisher, Waltham, MA, USA) according to the manufacturer’s instructions, yielding 936 ng (PMG) and 432 ng (AMG) mRNA. Nucleic acid concentrations were quantified by absorption at 260 nm (A_260_) on a Nanodrop 2000 spectrophotometer (Thermo Fisher Scientific, Waltham, MA, USA). Construction and sequencing of RNA-Seq libraries were performed by the Sequencing Facility at the Institute for Molecular Bioscience, The University of Queensland, Australia. TruSeq libraries for each of the PMG and AMG were constructed from ~300 ng mRNA. The multiplexed libraries were then sequenced as a part of a high-output run on an HiSeq 2500 (Illumina, San Diego, CA, USA), yielding 42,324,442 and 67,317,010 150 bp paired-end reads for the PMG and AMG, respectively.

Combined transcriptomes of the PMG and AMG were assembled using Trinity 2.2.0 [43] and CLC Genomics Workbench 8.0.2 software (CLC Bio, Aarhus, Denmark). Sequences were trimmed with default settings on Trimmomatic before Trinity assembly, or to a quality rate of 0.01 with maximum ambiguous bases = 2 in CLC Genomics Workbench before CLC assembly. The number of assembled contigs in the Trinity assembly and the CLC assemblies with a k-mer size of 64, 54, 44, 34, 29, and 24 was 106,023; 77,935; 74,707; 70,663; 66,109; 63,640; and 60,574, respectively. To produce a database for MS-based protein identification, these assemblies were pooled into a single file, from which open reading frames encoding polypeptides >30 amino acid residues were extracted using TransDecoder. Removing the redundant sequences using CD-HIT (identity threshold of 100%, word size 8) generated 171,393 possible amino acid sequences. These sequences were used as a database for MS identification of proteins, together with a database of 159 common MS contaminants.

Although the pooled assemblies clustered at 100% were ideal for polypeptide identification, they were unsuitable for examination of transcript abundance due to their high redundancy. Therefore, we produced transcript abundance measures in the following way: (1) We generated a smaller database containing just the ORFs encoding venom polypeptides detected by mass spectrometry (which also have been filtered for redundancy using the ProtGroup algorithm; see Section 4.4); (2) CLC Genomics Workbench was used to map the quality-filtered reads from either the PMG or the AMG (using similarity threshold = 0.9, minimum overlap = 0.8, and maximum hit sequences = 1) against this smaller database. ORF length was then used to convert to fragments per kilobase (FPK); (3) To convert to TPM, we calculated the total FPK (ΣFPK) for the whole transcriptome from two of our individual assemblies, the Trinity assembly and the CLC assembly using k-mer size 64. Each assembly was first clustered to reduce errors resulting from redundancy using CD-HIT (identity threshold 90%, word size 8). Filtered reads obtained from each of the PMG and AMG were then mapped separately against each of the two clustered transcriptomes, using the same parameters as above. ΣFPK was calculated for each of the Trinity and CLC datasets, and the mean of the two values was used for normalisation to TPM values, resulting in a single TPM value for each contig in each of the PMG and AMG.

We also tested if our TPM values might be biased due to different overall RNA expression levels between the two glands using the trimmed mean of M-values (TMM) method [44]. Contigs to which zero fragments were mapped from either of the AMG and PMG dataset were first filtered out. A-values were calculated for each contig, and M-values were calculated for AMG expression with reference to the PMG:

$$M\;=\;log_{2}\left(\frac{\mathrm{fragments}\;\mathrm{mapped}\;\mathrm{to}\;\mathrm{contig}}{\mathrm{total}\;\mathrm{fragments}\;\mathrm{mapped}}\right)$$

$$A\;=\frac{1}{2}\;log_{2}\left(\frac{\mathrm{AMG}\;\mathrm{fragments}\;\mathrm{mapped}\;\mathrm{to}\;\mathrm{contig}}{\mathrm{total}\;\mathrm{AMG}\;\mathrm{fragments}\;\mathrm{mapped}}\times \;\frac{\mathrm{PMG}\;\mathrm{fragments}\;\mathrm{mapped}\;\mathrm{to}\;\mathrm{contig}}{\mathrm{total}\;\mathrm{PMG}\;\mathrm{fragments}\;\mathrm{mapped}}\right)$$

The top and bottom 30% of M-values and the top and bottom 5% A values were then trimmed (default parameters according to [44]). Using the contigs remaining, the normalisation factor was calculated as:

$$Normalisation\;factor\;=\;\frac{\sum wM}{\sum w}$$

where

$$w\;=\;\frac{\mathrm{total}\;\mathrm{AMG}\;\mathrm{fragments}\;\mathrm{mapped}\;-\;\mathrm{AMG}\;\mathrm{fragments}\;\mathrm{mapped}\;\mathrm{to}\;\mathrm{contig}}{\mathrm{total}\;\mathrm{AMG}\;\mathrm{fragments}\;\mathrm{mapped}\;\times \;\mathrm{AMG}\;\mathrm{fragments}\;\mathrm{mapped}\;\mathrm{to}\;\mathrm{contig}}\;\;+\;\frac{\mathrm{total}\;\mathrm{PMG}\;\mathrm{fragments}\;\mathrm{mapped}\;-\;\mathrm{PMG}\;\mathrm{fragments}\;\mathrm{mapped}\;\mathrm{to}\;\mathrm{contig}}{\mathrm{total}\;\mathrm{PMG}\;\mathrm{fragments}\;\mathrm{mapped}\;\times \;\mathrm{PMG}\;\mathrm{fragments}\;\mathrm{mapped}\;\mathrm{to}\;\mathrm{contig}}$$

However, since we found evidence for only a very small effect (scaling factor for AMG with reference to PMG = 0.98), we proceeded to explore the TPM values obtained.

### 4.3. Electrophoresis

Approximately 400 µg of venom (quantity estimated using A_280_ measured on a Nanodrop 2000 spectrophotometer (Thermo Fisher Scientific, Waltham, MA, USA) was lyophilised, then re-suspended in 125 µL of resuspension buffer (8 M urea, 4% CHAPS) supplemented with 10 mM DTT and 1% ampholyte solution (#17-6000-88; GE Healthcare, Little Chalfont, UK). The sample was then absorbed into a Readystrip immobilized pH gradient strip (pH 3–10, nonlinear, #163-2005, Bio-Rad, Hercules, CA, USA) for 16 h. Isoelectric focusing then was conducted under mineral oil on an Ettan IPGphor3 isoelectric focuser (GE Healthcare, Chicago, IL, USA) using a program of 5900 total Vh. The strip was then incubated in reducing reagent (50 mM Tris-HCl, pH 8.8, 6 M urea, 2% SDS, 30% glycerol, 1.5% DTT) for 10 min, followed by alkylation reagent (50 mM Tris-HCl, pH 8.8, 6 M urea, 2% SDS, 30% glycerol, 2% iodoacetamide) for 20 min. Second dimension SDS-PAGE was then performed using a 15% Tris-glycine polyacrylamide gel alongside a Precision Plus ladder (#161037, Bio-Rad, Hercules, CA, USA), then the gel was stained with colloidal Coomassie stain [45].

### 4.4. Proteomics 

LC–MS/MS of whole venom samples and 2D-SDS-PAGE spots was performed as previously described [14,15]. Reduced, alkylated and trypsinised samples were prepared by diluting 5–50 µg venom (estimated from A_280_ measured on a Nanodrop 2000 spectrophotometer (Thermo Fisher Scientific, Waltham, MA, USA) in a reducing/alkylating solution (1% 2-iodoethanol, 0.25% triethylphosphine, 48.75% acetonitrile, 50 mM ammonium bicarbonate pH 11.0) and incubating the sample for 1 h at 37 °C. Samples then were dried by vacuum centrifugation and reconstituted in a digestion reagent (20 ng/µL sequencing grade trypsin, (#7575, Sigma Aldrich, St. Louis, CA, USA) in 40 mM ammonium bicarbonate pH 8.0, 10% acetonitrile) before quenching in an extraction reagent (50% acetonitrile, 5% formic acid), vacuum centrifugation, and reconstitution in 1% formic acid. Reduced and alkylated (but not trypsinised) and native MS samples also were generated by omitting the digestion step in the above protocol. Gel spots were excised with a scalpel, incubated for 2 h at 37 °C in a reduction/alkylation reagent, then incubated for 4–16 h at 37 °C in 5–20 µL of a digestion reagent. Samples where then incubated for 30 min at room temperature in an extraction reagent with occasional vortexing.

LC-MS/MS was performed by loading protein samples on a Zorbax 300SB-C18 column (#858750–902, Agilent, Santa Clara, CA, USA) and eluting them over 75 min using a gradient of 1–40% solvent B (90% acetonitrile and 0.1% formic acid) in solvent A (0.1% formic acid) at a flow rate of 0.2 mL/min on a Nexera X2 LC system (Shimadzu, Kyoto, Japan). The LC outflow was coupled to a 5600 Triple TOF mass spectrometer (SCIEX, Framingham, MA, USA) equipped with a Turbo V ion source. MS1 scans were collected between 350 and 2200 *m*/*z*, and precursor ions in the range *m*/*z* 350–1500 with a charge from +2 to +5 and signal >100 counts/s were selected for analysis, excluding isotopes within 2 Da. MS/MS scans were acquired with an accumulation time of 250 ms and a cycle time of 4 s. The ‘Rolling collision energy’ option was selected in Analyst software, allowing collision energy to be varied dynamically based on *m*/*z* and *z* of the precursor ion. Up to 20 similar MS/MS spectra over an *m*/*z* range 80–1500 were pooled from precursor ions differing by <0.1 Da. The resulting mass spectra in WIFF format then were compared to our MS amino acid sequence database generated from RNA-Seq data using a Paragon 4.0.0.0 algorithm in ProteinPilot 4.0.8085 software (SCIEX, Framingham, MA, USA). Minimum criteria used to identify proteins from venom samples were three or more peptides observed with >95% confidence (*p*  >  95%), or one or more peptide *p*  >  95% plus a secretion signal sequence with *D*-score >0.7 according to SignalP 4.1 [46]. Some incomplete ORFs were re-analysed by mapping trimmed reads against them using Geneious software. The final venom proteome amino acid sequences and ORFs were submitted to GenBank (accessions MN208278–MN208442).

### 4.5. Calcium Imaging of Mouse Sensory Neurons

DRG from 4-week-old male C57BL/6 mice were dissociated then plated in DMEM (Invitrogen) containing 10% foetal bovine serum (FBS) (Assaymatrix, Melbourne, Australia) and penicillin/streptomycin (Gibco, Waltham, MA, USA) on a 96-well poly-D-lysine-coated culture plate (Corning, New York, NY, USA) and maintained overnight. Cells were loaded with a Fluo-4 AM calcium indicator according to the manufacturer’s instructions (Thermo Fisher Scientific. Waltham, MA, USA). Following loading (30 min at 37 °C, then 30 min at room temperature), the dye-containing solution was replaced with an assay solution (Hanks’ balanced salt solution, 20 mM HEPES). Fluorescence corresponding to [Ca^2+^]*_i_* of 100–150 DRG cells was monitored in parallel using a Ti-E deconvolution inverted microscope (Nikon, Tokyo, Japan), equipped with a Spectra LED lightsource (Lumencore, Beaverton, OR, USA. Images were acquired at 20× objective at one frame per second (excitation 485 nm, emission 521 nm). Baseline fluorescence was monitored for 30 s. The assay solution was replaced with an assay solution (negative control) at 30 s, and at 60 s with *P. rhadamanthus* venom (100 ng/µL in assay solution). Experiments involving the use of mouse tissue were approved by The University of Queensland animal ethics committee (TRI/IMB/093/17).

### 4.6. Insecticidal Assays

The toxicity of *P. rhadamanthus* venom was evaluated by injection into female fruit flies (*D. melanogaster*) four to six days post-emergence. Female flies were used to reduce variability due to size, and because their larger size makes injection easier. A group of eight female flies were assayed in triplicate for each dose tested. Injection needles were formed by pulling glass capillary tubes (#3-000-203-G/X, Drummond, Birmingham, AL, USA) on a micropipette puller (Sutter Instrument Co. Model P-97) and trimming the fine tip manually using tweezers. Needles then were filled with mineral oil, connected to a Nanoliter 2000 microinjector (Kanetec, Bensenville, IL USA) equipped with a foot pedal, and ~1 µL of venom or water aspirated. Each group of flies were immobilised by cooling the 5 mL tube in which they were contained for approximately 2 min on ice, and then decanted on the top of the ‘injection stage’, a petri dish filled with ice. Under a dissecting microscope, each fly was carefully impaled on the lateral thorax behind the wing, and 50.6 nL of diluted venom or water was injected. Subsequent to injection, each group of flies were returned to their 5 mL plastic tube at room temperature and their behaviour observed.

### 4.7. Data Processing

Protein quantification measures were obtained for each protein detected in LC-MS/MS datasets by averaging the three highest precursor count values for peptides derived from that protein, consistent with the ‘best flier’ hypothesis [15,47]. Statistical tests were performed using Rstudio software or the RealStatistics package in Microsoft Excel (Microsoft, Redmond, WA, USA). Plots were prepared using IgorPro (Wavemetrics, Lake Oswego, OR, USA and RStudio (RStudio Inc., Boston, MA, USA).

## Figures and Tables

**Table 1:** Mature peptides detected by LC-MS/MS analysis of native and reduced/alkylated venom samples. Masses shown are monoisotopic.

Amino Acid Sequence	Precursor	Putative Fold	No. Cys	Theoretical Native Mass (Da)	Observed Native Mass (Da)	Theoretical RA^a^ Mass (Da)	Observed RA Mass (Da)
DEKDCIARGQKCVGENKPCCKGTTCMYYANRCVGV	U-RDTX ^b^-Pr1a	ICK ^c^	6	3835.69	3835.62	4105.89	4105.81
TIEKSCKPGTTFKHKDGCNTCKCSDDGKNALCTSKLCL	U-RDTX-Pr11a.2	Pacifastin	6	4070.88	4070.81	4341.09	4340.99
TMKQSCKPGATFKHKDGCNTCKCSDDGKSARCTARLCW	U-RDTX-Pr10a.2	Pacifastin	6	4158.86	4158.78	4429.06	4428.96
EEHGCIPPFQPCEGVNSRCCGLYVCFNKICLATP	U-RDTX-Pr2a	ICK	6	3763.65 ^b^	3763.59 ^d^	4033.86 ^d^	4033.77 ^b^
SRACSKPGQTVLAPDGCNHCRCSEKGILMACTKMMCPPR	U-RDTX-Pr11a.1	Pacifastin	6	4172.89	4172.82	4443.10	4442.99
HGCIPPFQPCEGVNSRCCGLYVCFNKICLATP	U-RDTX-Pr2b	ICK	6	3505.57 ^b^	3505.51 ^d^	3775.77 ^d^	3775.68 ^b^
GGCIQRYGKCSTENSNCCAPSECYFSFNQCF	U-RDTX-Pr7a	ICK	6	3439.33	3439.26	3709.53	3709.43
CIPAANPCRGNAKCCGNYVCKNGRCLPRS	U-RDTX-Pr5a	ICK	6	3061.39	3061.31	3331.59	3331.52
MMPVCFEGEKLNKDQTKCIKA	U-RDTX-Pr9a	Unknown	2	2410.16	2410.13	2500.23	2500.19
GEDVCIPSGQKCGPYMNMGCCKGLVCMSYAARCVSMGGIPR	U-RDTX-Pr4a	ICK	6	4264.84	4264.77	4535.05	4534.09
MDCKPGKKFKIDCNTCICSKEGKAAACTQKLCLK	U-RDTX-Pr21.2	Pacifastin	6	3699.79	3699.72	3970.00	-
CRPGALTVAPDGCNMCTCLSNGKLGRCTHDLICPPR	U-RDTX-Pr12a.1	Pacifastin	6	3765.73	-	4035.93	4035.85

Cysteine residues inferred by measured masses to form intrachain disulfide bonds are shown highlighted in grey. ^a^ RA, reduced and alkylated with 2-iodoethanol (addition of 45.034 Da per half-cystine). ^b^ RDTX, reduvitoxin. ^c^ ICK, inhibitor cystine knot. ^d^ Observed MS1 and MS2 spectra for Pr2a and Pr2b suggest that K28 of Pr2a (K26 of Pr2b) is carbamylated, but this assignation is putative.

**Table 2:** Major spots with unambiguous LC-MS/MS identifications.

Spot	Protein	Theoretical pI	Observed pI	Theoretical Mass (kDa)	Observed Mass (kDa)	ProteinPilot Score ^a^
5	Gelsolin 1	7.2	7.1	39.3	49	60.9
17	C1A protease 1	8.0	6.4	35.2	35	28.8
27	Gelsolin 1, subdomain 1	5.2	4.8	14.1	14	15.0
28	Gelsolin 1, subdomain 1	5.2	5.2	14.1	14	20.0
29	CUB domain protein 2	5.0	4.7	12.6	16	4.0
30	CUB domain protein 1	5.0	5.1	12.1	15	18.1
34	Family 4 protein 1	8.9	6.4	17.1	16	32.0
35	Gelsolin 1, subdomain 1	5.2	5.6	14.1	15	26.3
43/82/126	Redulysins, N-terminal fragments	Nd ^b^	6–10	nd	<10	14.5–18.9
80	Family 1 protein 1	7.9	7.8	13.8	13	21.3
88	Family 1 protein 2	9.0	8.9	14.8	14	56.0
93	Redulysins 2,3,7, active form	9.1–9.6	7–10	21.0–23.5	18–22	>15
96	Redulysin 2, active form	9.3	9.1	21.5	20	54.6
97	Gelsolin 1, subdomains 2 and 3	9.0	9.0	25.2	25	69.9
110	Serpin 1	5.5	6.4	42.6	35	36.6
various	S1 proteases	nd	4–10	nd	23–50	nd

^a^ ProteinPilot scores >1.3 corresponds to 95% confidence at the protein level. ^b^ nd, not determined.

## Supplementary Materials

The following are available online at https://www.mdpi.com/2072-6651/11/11/673/s1, Supplementary Data S1: *Platymeris rhadamanthus* proteome sequences and annotations, Supplementary Data S2: Transcript abundances of venom-protein-encoding and other genes, Supplementary Data S3: Putative orthologous gene products in the venoms of *Platymeris rhadamanthus* and *Pristhesancus plagipennis*; Figure S1: Alignment of *Platymeris rhadamanthus* venom toxins with previously reported venom proteins of reduviine and triatomine reduviids.
