# Supplementary material for: Missiles of Mass Disruption: Composition and Glandular Origin of Venom Used as a Projectile Defensive Weapon by the Assassin Bug Platymeris rhadamanthus
Source: Toxins (Basel). 2019 Nov 18;11(11):673. doi: 10.3390/toxins11110673 (PMC6891600; doi:10.3390/toxins11110673)
Supplement: Supplementary file 1 [file toxins-11-00673-s001.zip › toxins-612131 -SI.docx]

Supplementary Materials: Missiles of Mass Disruption: Composition and Glandular Origin of Venom Used as a Projectile Defensive Weapon by the Assassin Bug *Platymeris rhadamanthus*

Andrew A. Walker, Samuel D. Robinson, Eivind A. B. Undheim, Jiayi Jin, Xiao Han, Bryan G. Fry, Irina Vetter and Glenn F. King


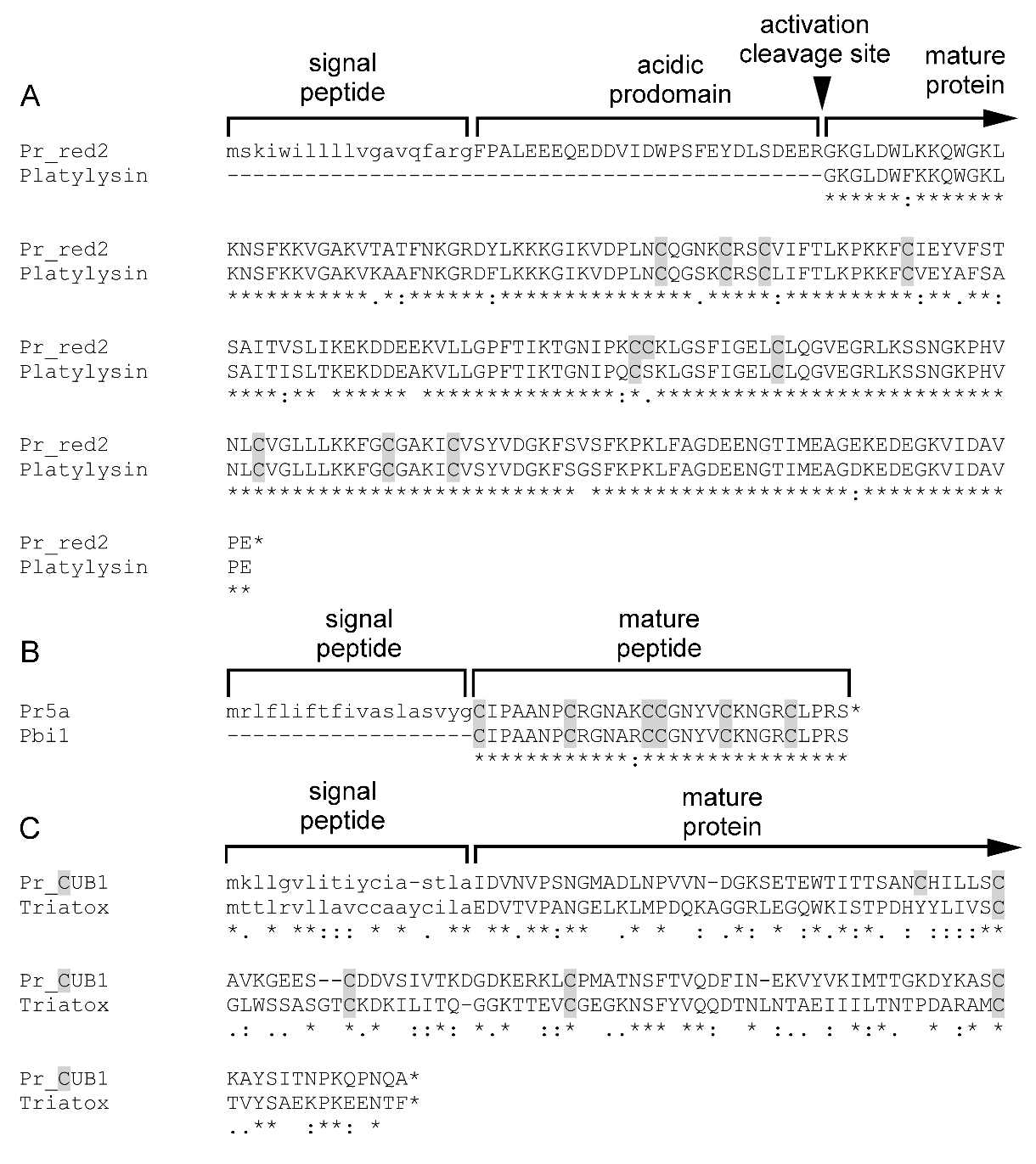


**Figure S1:** Alignment of *Platymeris rhadamanthus* venom toxins with previously reported venom proteins of reduviine and triatomine reduviids. (**A**) Redulysin 2 from *Platymeris rhadamanthus* (Pr_red2) with Platylysin from *Platymeris biguttatus*. (**B**) *Platymeris rhadamanthus* peptide Pr5a with *Platymeris biguttatus* peptide Pbi1a. (**C**) CUB domain protein 1 from *Platymeris rhadamanthus* (Pr_CUB1) with Triatox from *Triatoma infestans*.
